# Evaluation of the Efficacy of Flupyradifurone against *Bemisia tabaci* on Cassava in Tanzania

**DOI:** 10.3390/insects13100920

**Published:** 2022-10-12

**Authors:** Khamis A. Issa, Everlyne N. Wosula, Flora Stephano, James P. Legg

**Affiliations:** 1 International Institute of Tropical Agriculture, Dar es Salaam P.O. Box 34441, Tanzania; 2 Department of Zoology and Wildlife Conservation, University of Dar es Salaam, Dar es Salaam P.O. Box 35064, Tanzania

**Keywords:** flupyradifurone, *Bemisia tabaci*, cutting dip, CMD, CBSD, IPM

## Abstract

**Simple Summary:**

Cassava-colonizing *Bemisia tabaci* whitefly negatively impact cassava production in sub-Saharan Africa through transmission of viruses that cause cassava mosaic disease (CMD) and cassava brown streak disease (CBSD). This study evaluated the efficacy of a novel insecticide flupyradifurone against this whitefly pest through standard spraying and cutting dip methods. The results from the laboratory, screenhouse and field experiments consistently showed flupyradifurone is effective at reducing whiteflies compared to control treatments. A single cutting dip using this insecticide was highly effective at reducing whiteflies under field conditions and, therefore, should be considered as an alternative to insecticide spraying in cassava systems. Additionally, whiteflies were least abundant during the long rainy season (Masika) and on cassava variety Mkuranga1.

**Abstract:**

A novel butenolide insecticide—flupyradifurone (Sivanto SL 200)—was evaluated for efficacy against cassava-colonizing *Bemisia tabaci* whitefly under laboratory, screenhouse and field conditions. LC50 values from leaf disc spray assays were comparable for both flupyradifurone (12.7 g a.i/100 L) and imidacloprid (12.6 g a.i/100 L). Both insecticides caused high levels of adult whitefly mortality in leaf disc and leaf dip assays when compared to untreated controls. In screenhouse-based trials, longer soaking (60 min) with flupyradifurone or imidacloprid was more effective than shorter soaking durations (15 or 30 min). In field spraying experiments, flupyradifurone significantly reduced whiteflies, and both insecticides demonstrated powerful knockdown effects on whitefly adult abundances over a period up to 24 h. Single cutting dip application of flupyradifurone reduced whitefly adult abundance by 2 to 6 times, and nymphs by 2 to 13 times. Lower whitefly abundances resulting from insecticide application reduced the incidence of CMD or CBSD. In addition, in field experiments, whiteflies were fewer during the long rainy season (Masika) and on cassava variety Mkuranga1. The findings from this study demonstrate that cutting dips with flupyradifurone could be incorporated as a management tactic against cassava whiteflies. This would ideally be combined in an IPM strategy with other cassava virus and virus vector management tactics including host-plant resistance, phytosanitation and the use of clean seed.

## 1. Introduction

The whitefly *Bemisia tabaci* (Gennadius) (Hemiptera, Aleyrodidae), is one of the most economically damaging pest species of crop plants in Africa. It causes physical damage as well as transmitting many plant viruses [1,2]. Besides its importance with regard to cassava, the species complex is well known across the world as one of the most important global pests and has been reported on more than 1000 species belonging to over 100 plant families [3].

Cassava (*Manihot esculenta* Crantz) is an important subsistence crop throughout much of sub-Saharan African and is perceived as Africa’s future food security hope, due to its anticipated resilience to the effects of climate change [4]. However, pests and plant diseases reduce cassava yields substantially, posing a threat to food security throughout the developing world [5]. Two viral diseases that are spread by whiteflies cause important economic losses to cassava production. These are cassava mosaic disease (CMD) and cassava brown streak disease (CBSD) [6]. CMD is caused by cassava mosaic begomoviruses (CMBs) [7] which are transmitted persistently by *B. tabaci* [8]. CMD occurs in all cassava-growing parts of sub-Saharan Africa [9]. CBSD is caused by cassava brown streak ipomoviruses (CBSIs) [10], which *B. tabaci* transmits in a semi-persistent manner [11,12]. Although the distribution of CBSD is confined to coastal East Africa and parts of Central Africa, it is spreading rapidly through the Democratic Republic of Congo and poses a great threat to cassava production zones of global significance in West Africa [13,14]. CMD causes losses through reducing plant growth that results from the extensive chlorosis that the viruses cause [9]. CBSD infection usually causes mild vein-associated chlorosis on lower leaves and can cause stem die-back in severe cases. However, the most common cause of yield loss is the dry, brown, necrotic rot that occurs in the maturing tuberous roots of infected plants [15]. Overall, these viruses affect more than half of all cassava plants in sub-Saharan Africa and cause annual losses of more than USD 1 billion [16,17].

Different methods have been used worldwide to control whitefly, for example, the use of insecticides and biocontrol using natural enemies such as parasitoids. The most widely used conventional insecticides are the neonicotinoids such as imidacloprid. Imidacloprid has been used successfully to manage *B. tabaci* and whitefly-borne geminiviruses on tomato in south Florida and elsewhere [18,19]. It is relatively effective in controlling *B. tabaci* on cassava, when applied either as a soil drench or foliar spray. However, the overuse of neonicotinoids presents a strong risk of cross-resistance between various chemicals of this group and threatens their effectiveness [20,21,22]. Increasing numbers of resistance cases to neonicotinoids (e.g., imidacloprid and thiamethoxam) have been documented [23,24]. Recently, it has been reported that flupyradifurone, a new insecticide that belongs to the butenolide class, is effective in reducing levels of virus transmission by *B. tabaci* MED species in tomato [25]. Flupyradifurone, registered name Sivanto (SL 200), belongs to Bayer Crop Science’s own chemical class of butenolides (Bayer AG, Monheim, Germany). It is a systemic insecticide, with flexible modes of application, and is mainly intended for control of sucking pests such as aphids, hoppers and whiteflies. Flupyradifurone’s mode of action is by binding to the insect’s nicotinic acetylcholine receptors (nACHRs), disrupting the nervous system, and thereby causing muscle paralysis and subsequent death of the treated insects [26]. In practical conditions, flupyradifurone can be considered safe to most beneficial insects including pollinators after having passed tests for its side effects on beneficial arthropods. It is currently approved and registered in Europe by the European commission in accordance with regulation EC 1107/2009, while in East Africa, it is registered in Kenya [27] and Tanzania [28].

A study was carried out by [25] to test flupyradifurone for its efficacy in controlling whiteflies and their transmission of tomato yellow leaf curl virus in comparison to a neonicotinoid insecticide, thiamethoxam. After foliar application at recommended label rates under greenhouse conditions, it suppressed virus transmission by 85%, while levels of suppression after thiamethoxam treatments were just 25% and significantly lower in untreated plots, where 100% of the plants were infected by virus [25]. A study carried out to evaluate field-collected populations of *B. tabaci* MEAM1 response to flupyradifurone in comparison to imidacloprid, thiamethoxam and dinotefuran showed some level of cross-resistance [29]. *Bemisia tabaci* is a cryptic species complex with the cassava-colonizing type designated based on mitochondrial DNA cytochrome oxidase I (*COI*) sequencing as sub-Saharan Africa (SSA), which consists of five groups (SSA1-5), with SSA1 divided into sub groups (SSA1-SG1 to SG5) [30,31]. Recently, six major haplogroups of cassava *B. tabaci* were defined (SSA-ECA, SSA-ESA, SSA-CA, SSA-WA, SSA2, SSA4) based on the much more genetically informative SNP genotyping [32]. *Bemisia tabaci* cryptic species are known to respond differently to insecticides, with some such as MEAM1 and MED developing resistance to a wide range of current chemistries [33]. The cryptic species that is predominant in the study area is haplogroup SSA-ESA (mitotype SSA1-SG3) [31,32]. In cassava, one recent study reported that imidacloprid applied through cutting dips at planting followed by varying spray intervals was effective at reducing whitefly populations, and significantly reducing cassava yield loss by up to 60% [34]. In spite of this result, there are currently no effective management strategies being applied for *B. tabaci* control on cassava in sub-Saharan Africa. A study was therefore conducted to assess the efficacy of the novel product flupyradifurone with the aim of determining whether or not this novel ‘soft chemistry’ insecticide might have the potential to be incorporated into integrated pest and disease management strategies for whitefly and whitefly-transmitted virus control in cassava. The findings from this study should provide information on the efficacy of flupyradifurone against cassava whiteflies but also, more specifically, on the *B. tabaci* haplogroup SSA-ESA.

## 2. Materials and Methods

### 2.1. Plant Materials, Whitefly Colony and Chemical Insecticides

Cassava virus-free planting materials of varieties Albert, Kiroba and Mkuranga1 were collected from clean seed sites at Makutopora in Dodoma Region and at Mwele in Tanga Region, Tanzania. Leaf materials from all stems were tested for cassava brown streak ipomoviruses (CBSIs) using standard real-time PCR diagnostics based on TaqMan chemistry [35] prior to planting. The *Bemisia tabaci* whitefly haplogroup used in this study was sub-Saharan Africa—East and Southern Africa (SSA-ESA—equivalent to mitotype SSA1-SG3) [32]. The whiteflies were collected from cassava plants at Chambezi in Bagamoyo District, Coast Region, Tanzania, and introduced to potted cassava plants placed in 50 × 50 × 100 cm netted cages. The plants were grown in 7.5 L pots containing a mixture of soil and farmyard manure at a 4:1 ratio, and the whitefly colonies were reared on the cassava plants in the screenhouse at 25–35 °C and 65–75% RH. Whiteflies were transferred to fresh one-month-old cassava plants at intervals of four to six weeks to maintain the colonies. The insecticide flupyradifurone (Sivanto^®^ 200SL) was provided by Bayer AG (Monheim, Germany). The insecticide imidacloprid (Septer ^®^ 200SL) was acquired locally (Balton, Tanzania). Laboratory, screenhouse and field experiments were designed to evaluate flupyradifurone and compare it with imidacloprid and water-only controls.

### 2.2. Laboratory and Screenhouse Experiments

#### 2.2.1. Leaf Disc Assay

In the spray assays, cassava leaf portions obtained from the second fully expanded leaf of cassava were used. They were cut with a scalpel into small portions (1 cm × 1.5 cm), dipped in a 2% hypochlorite solution for two minutes and then rinsed twice in distilled water before being lightly dried with a tissue. The leaf portions were then placed on tissue paper and mist-sprayed with insecticide solution using a 2 mL fingertip sprayer to cover the entire leaf surface. Flupyradifurone and imidacloprid each had six different concentrations: 0.33 g active ingredient (a.i)/100 L (3.3 ppm), 1 g a.i/100 L (10 ppm), 3 g a.i/100 L (30 ppm), 15 g a.i/100 L (150 ppm), 45 g a.i/100 L (450 ppm), and 135 g a.i/100 L (1350 ppm). After drying, the leaf portions were placed on a thin layer of agar (0.5% water agar) in a Petri dish (5.5 cm × 1.0 cm) with the adaxial surface laid on top of the agar. This positioning of the leaf portions was performed to mimic the normal orientation of whitefly interaction with an intact plant. Thirty whiteflies were introduced through a small hole in the lid of the Petri dish using a pipette tip mouth aspirator, and the hole was plugged using Parafilm. The dishes were placed upside down on a laboratory bench such that the leaf portions and adult whiteflies feeding on them were in the natural orientation (whiteflies feed on the undersides of leaves). Mortality of whiteflies was recorded daily over a period of 5 days, and the experiment was replicated three times.

#### 2.2.2. Leaf Dip Assay

Cassava leaves were obtained from 4–6-week-old potted plants (2nd–3rd fully opened leaves from the top). The leaf petioles were immersed in insecticide solution of flupyradifurone (100 g a.i/100 L) or imidacloprid (200 g a.i/100 L) overnight. Leaflets were then cut and placed in 2 mL Eppendorf vials containing distilled water that were sealed with Parafilm. Twenty whiteflies that had emerged within four days were aspirated into glass vials (7.5 cm × 2.5 cm diameter—Watkins and Doncaster, UK). The Eppendorf and glass vials containing leaflets and whiteflies (20 per vial), respectively, were held in round fit holes in transparent plastic containers (7.5 cm × 10 cm diameter) with lids placed upside down. Whiteflies were confined by covering the plastic containers with perforated bread bags (25 cm × 20 cm) fastened at the bottom end with a rubber band. This experiment consisted of 5 replicates per treatment, and it was conducted 2 times to give a total of 10 replicates per treatment. Dead whiteflies were recorded at intervals of 3 h, 24 h, 48 h, 72 h and 96 h.

#### 2.2.3. Cutting Dip Assay

Flupyradifurone was tested for its efficacy in controlling *B. tabaci* through dipping cuttings in treatment solution. Imidacloprid and water were used as controls. All these experiments were performed in a screenhouse at IITA-Tanzania, Dar es Salaam, using the whitefly-preferred cassava variety—Albert. Cassava cuttings were dipped into solutions of flupyradifurone (100 g a.i/100 L), imidacloprid (200 g a.i/100 L) or water. Twelve pots for each treatment were placed in three insect-proof cages (four pots per cage). This meant that there were three cage replicates for each treatment. After the cuttings had sprouted, 120 adult whiteflies were introduced into each cage, and numbers of live whiteflies were recorded for a period of five days. The mortality was recorded by counting the number of live adult whiteflies remaining on the leaves, hence subtracting from the total whiteflies introduced before observation. Three experiments with three different dipping times were conducted to compare the effect of the dipping duration. The three durations tested were 15, 30 and 60 min.

### 2.3. Field Experiments

#### 2.3.1. Foliar Insecticide Application

Field trials were conducted at Chambezi Research Station. This location has tropical weather conditions with a bi-modal rainfall pattern that comprises a long rainy season (Masika) between March and June and a short rainy season (Vuli) between October and December [36]. This location was selected in view of the high incidences of CMD and CBSD and abundant populations of the whitefly vector—*B. tabaci*. A field experiment was carried out for two planting seasons, including Vuli 2016/17 (planted 13 October 2016) and Masika 2017/18 (planted 8 March 2017). The design was a randomized split plot with insecticide treatments allocated as the main plots and cassava varieties allocated to split plots (42 plants per split plot). Sorghum barriers were planted between plots to minimize interference between the different spray treatments, and each main plot had three split plots. The size of the main plots was 18 m by 7 m, and the distance between main plots was 10 m. When edge rows were excluded, there were 20 plants in the ‘net plot’ for each split plot. Insecticides were applied as foliar applications with the flupyradifurone manufacturer-recommended dosage of 110 g a.i/ha at sprouting, 3 weeks after planting (3 WAP), 6 WAP and 12 WAP. Imidacloprid was applied using the local recommended dose of 200 g a.i/ha and with a spray interval the same as that of flupyradifurone. In addition, there were untreated plots that were left as negative controls. For each planting season, counts of *B. tabaci* whiteflies, and incidences (percentage symptomatic plants) of CMD and CBSD were conducted monthly, from 1 month after planting (MAP) to 6 MAP. *Bemisia tabaci* whitefly adults were counted on the top five leaves of the tallest shoot of each sampled plant. In addition, the relative ‘knockdown’ effects of flupyradifurone and imidacloprid were evaluated by recording adult whitefly numbers at 2 h, 6 h and 24 h after spraying at 3 WAP and 6 WAP during the Vuli season.

Harvesting was conducted to assess the impact of virus disease incidences and whitefly abundance on cassava tuberous root production. Yield (fresh tuberous root biomass) was recorded using the 20 net plot plants. Weights of individual roots (grams per plant) were recorded using a weighing balance (Electronic scale-SF 400).

#### 2.3.2. Cutting Dip Insecticide Application

To test the cutting dip method within a field setting, a randomized complete block design was employed. There were four blocks and two varieties—Albert and Mkuranga1. Varieties within each block were randomly assigned to insecticide treatments, which were flupyradifurone, imidacloprid, and the control, which comprised untreated cuttings. A total of 80 cassava cuttings for each cassava variety were tied together and dipped for 1 h in 40 L diluted insecticide solutions contained in plastic drums. After dipping, cuttings were planted at a spacing of 1 m × 1 m in field plots. Manufacturer recommended concentrations were used for the insecticides: 110 g a.i/ha for flupyradifurone and 200 g a.i/ha for imidacloprid. Split plots comprised 4 × 5 arrays of 20 plants for each variety/insecticide treatment combination, and 5 m gaps were left between blocks.

After sprouting, 10 plants were randomly selected in each split plot and tagged for whitefly and nymph counts, as well as monthly assessments of percentage of symptomatic CMD and CBSD from 1 MAP to 6 MAP. *Bemisia tabaci* whitefly adults were counted as described previously, and numbers of 4th instar *B. tabaci* nymphs were recorded on the 9th and 10th leaves, or the lowest two fully opened leaves where plants did not yet have ten leaves. After twelve months, the plants were harvested to measure the yield of tuberous roots for each plant.

### 2.4. Data Analysis

Laboratory assays: Mean percentages of whitefly mortality were subjected to analysis of variance (ANOVA) using PROC GLIMMIX (SAS version 9.4; SAS Institute, Cary, NC, USA) with the Gamma distribution. Probit analysis of mortality vs. concentration was conducted to estimate lethal concentrations (LC50) with their corresponding 95% confidence intervals (95% CI) using the PROC PROBIT statement in SAS. LC values were considered to be significantly different when their respective 95% CI did not overlap.

Screenhouse and field experiments: Whitefly and nymph counts as well as incidences of CMD and CBSD were subject to ANOVA using PROC GLIMMIX with the Poisson distribution. The LSMEANS statement was used to obtain least squares means and the Tukey–Kramer test at *p* = 0.05 was used for pairwise comparison of treatment means for insecticide, variety and insecticide and variety interaction. Treatment means and standard errors were obtained using the PROC MEANS statement in SAS. Hour, day, and month data points were all considered independently. Whiteflies were characteristically very mobile and not restricted to the repeatedly sampled plants during the period of data collection.

## 3. Results

### 3.1. Laboratory and Screenhouse Experiments

#### 3.1.1. Leaf Disc Assay

**Figure 1:**
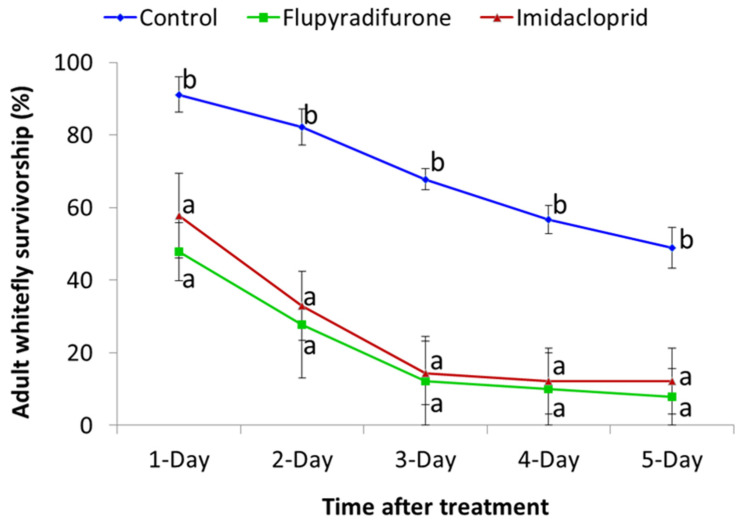
Survivorship of adult *B. tabaci* introduced to leaf disc assays and monitored at regular intervals up to five days after treatment with 15 g a.i/100 L imidacloprid and 15 g a.i/100 L flupyradifurone (Mean ± SE). Means with the same letter for a given time after treatment are not significantly different (*p* < 0.05, Tukey–Kramer test).

**Figure 2:**
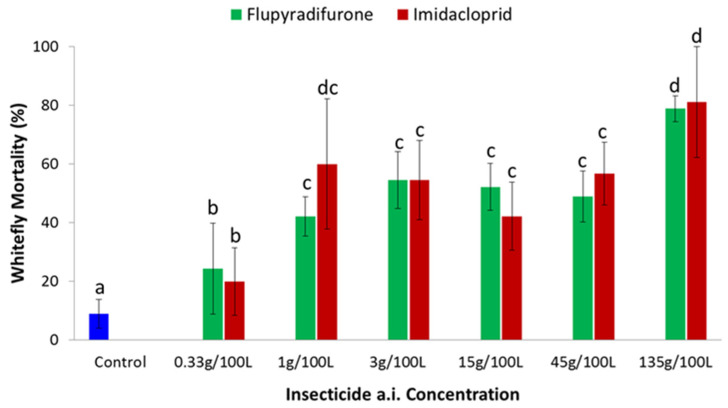
Mortality of adult *B. tabaci* 24 h post-treatment with varying concentrations of imidacloprid and flupyradifurone in comparison to control in the leaf disc assay (Means ± SE). Means with the same letter are not significantly different (*p* < 0.05, Tukey–Kramer test).

Leaf disc bioassays were used to evaluate efficacy of imidacloprid and flupyradifurone against cassava *B. tabaci*. Both insecticides caused increasing levels of mortality over time, and even at one day after treatment, the two products caused significantly higher (*p* < 0.05) whitefly mortality than the control (Figure 1). In general, whitefly mortality increased with increasing dose of imidacloprid and flupyradifurone (Figure 2), and greatest mortality for both products was recorded at the highest dose of 133 g a.i/100 L. Probit calculations were used to determine the LC_50_ values for the two products, using data for one day after treatment. The LC_50_ for imidacloprid was 12.6 g a.i/100 L, while that for flupyradifurone was 12.7 g a.i/100 L.

#### 3.1.2. Leaf Dip Assay

**Figure 3:**
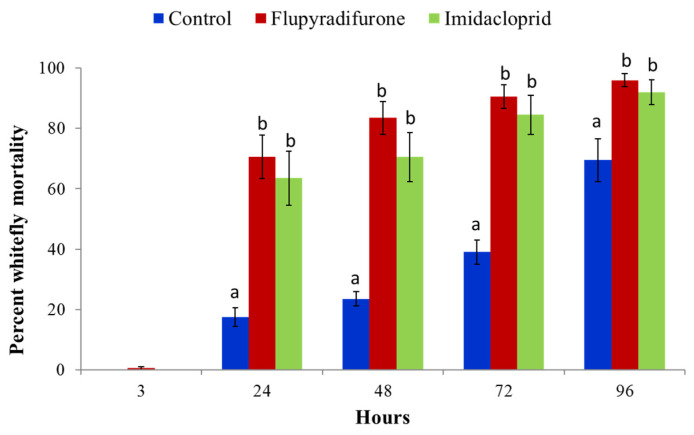
Percentage mortality of *B. tabaci* in the leaf dip assays treated with flupyradifurone and imidacloprid (Means ± SE). Mortality was recorded at 3, 24, 48, 72 and 96 h. Means with the same letter for a given time interval are not significantly different (*p* < 0.05, Tukey–Kramer test).

There were no significant differences between insecticide and control treatments at 3 h in the leaf dip assay, although differences were highly significant by 24 h (*p* < 0.0001), with mortalities of 70.5% for flupyradifurone and 63.5% for imidacloprid compared to 17.5% for the control (Figure 3). These significant differences were sustained through to the end of the experiment at 96 h. There were, however, no differences in mortality caused by the two insecticides at any of the time points, *p* > 0.05 (Figure 3).

#### 3.1.3. Cutting Dip Assay

The number of whiteflies on plants on day 1 with cuttings soaked for 60 min was significantly lower (*p* < 0.0001) for the flupyradifurone treatment compared to imidacloprid and the control. The plants with cuttings soaked for 60 min for both insecticides also had significantly fewer (*p* < 0.0001) whiteflies compared to those soaked for 15 and 30 min. On day 2, no whiteflies were present on plants soaked in flupyradifurone for 60 min, while those for 15 and 30 min had significantly fewer whiteflies compared to the control (*p* < 0.0001). There were significantly more whiteflies on plants from cuttings soaked for 60 min in imidacloprid compared to the flupyradifurone treatment, but fewer compared to the control (*p* < 0.0001). On days 3, 4 and 5, both flupyradifurone and imidacloprid treatments dipped for 15, 30 and 60 min had significantly fewer (*p* < 0.0001) whiteflies compared to the control. On day 5, there were no whiteflies present on plants from cuttings soaked with flupyradifurone for 30 or 60 min (Table 1). Overall, cutting dips with both insecticides were demonstrated to be highly effective in causing whitefly mortality, but the whitefly control effects of flupyradifurone were more rapid and of higher potency than those of imidacloprid.

### 3.2. Field Experiments

#### 3.2.1. Foliar Insecticide Application

Field spraying experiments were carried out for two seasons (Masika and Vuli) with three cassava varieties (Albert, Mkuranga1, Kiroba) and two insecticides (flupyradifurone and imidacloprid). The Masika season whitefly counts were very low, and no significant differences were observed between treatments, with most recordings having zero whiteflies. CMD and CBSD incidences during the Masika season were very low. CMD was only recorded in the susceptible variety Kiroba, where incidences at 6 MAP were 6.8% in the control and 9.5% for the imidacloprid treatment. There was significantly less CMD in the flupyradifurone treatment where no symptomatic plants were recorded. CBSD was present only in the susceptible variety Albert which had a significantly higher incidence at 6 MAP (*p* < 0.05) of 25.6% in the control compared to 8.6% in the flupyradifurone and 2.4% in the imidacloprid treatments. In the Vuli season, whiteflies were significantly fewer (*p* < 0.05) in number on the flupyradifurone and imidacloprid treatments compared to the control for all three varieties (Table 2). The whitefly control effects of the foliar applications were most apparent for the 2 MAP assessment, which was immediately after a spray treatment. Control effects of both insecticides were either marginally significant or not significant at all during later assessments at 3 MAP and 4 MAP. Whitefly abundances declined to 0 by 5 MAP and remained so for the final assessment (6 MAP).

**Figure 4:**
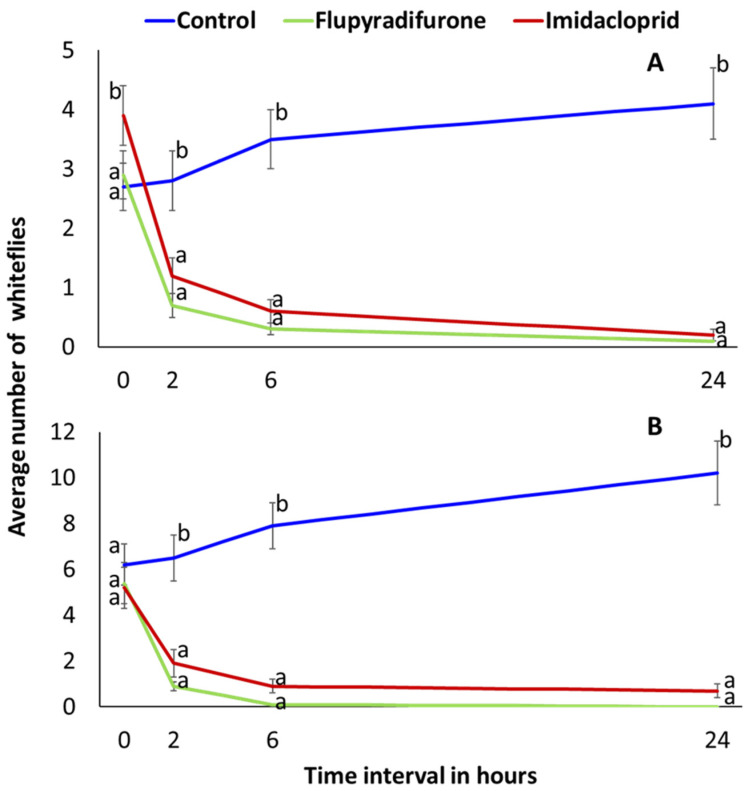
Insecticide (flupyradifurone and imidacloprid) knock-down effect on whitefly counts at 2 h, 6 h and 24 h after spraying at 3 weeks after planting (WAP) (**A**) and 6 WAP (**B**) (Means ± SE). Means with same letter within time interval are not significantly different (*p* < 0.05, Tukey–Kramer).

In the Vuli knockdown assessment performed at 3 WAP and 6 WAP, in which whiteflies were counted pre-spraying and subsequently at 2 h, 6 h and 24 h after spraying, there were significantly fewer (*p* < 0.0001) whiteflies in the insecticide treatments compared to the control (Figure 4). At 2 h post-spraying, whitefly adult abundance was reduced by 76% (3 MAP) and 83% (6 MAP) by flupyradifurone and 69% (3 MAP) and 63% (6 MAP) by imidacloprid. At 24 h post-spraying, whitefly adult abundance was reduced by 97% (3 MAP) and 100% (6 MAP) by flupyradifurone and by 95% (3 MAP) and 87% (6 MAP) in imidacloprid. In the control treatment, by contrast, abundance increased by 52% (3 MAP) and 65% (6 MAP) compared to the pre-spraying counts. Final CMD incidences recorded at 6 MAP in the Vuli trial differed between varieties but not between insecticide treatments (Table 3). Mean CMD incidences at 6 MAP were 10.3% for Albert, 23.3% for Kiroba and 0.6% for Mkuranga 1. No CBSD symptoms were recorded in Mkuranga1 throughout the six-month period of incidence recording, and consequently incidence was significantly less than for Albert (61.0%) and Kiroba (29.8%). For the most CBSD-susceptible variety (Albert), the final incidence at 6 MAP in the control treatment (85.4%) was significantly greater than incidences in the imidacloprid (59.6%) or flupyradifurone (38.0%) treatments (Table 4).

#### 3.2.2. Cutting Dip Insecticide Application

There were consistently fewer adult whiteflies in the insecticide treatments compared to the control throughout the six-month period of observations for plants that had sprouted from dipped cuttings (*p* < 0.05) (Table 5). Adult whitefly abundance was less for flupyradifurone-treated cuttings than it was for those treated with imidacloprid for both varieties at most time points. *B. tabaci* nymph counts were significantly lower (*p* < 0.05) on plants treated with flupyradifurone and imidacloprid compared to controls for all the six months except for 1 MAP with imidacloprid in Albert (Table 6). Flupyradifurone was significantly more effective (*p* < 0.05) than imidacloprid at reducing the number of nymphs for both varieties and at most time points from 1 MAP to 6 MAP (Table 6). The greatest percentage reductions in whitefly nymph abundance (comparing insecticide with control treatments) were at 4 MAP for both flupyradifurone (88.9% reduction for Albert; 91.7% reduction for Mkuranga1) and imidacloprid (71.7% reduction for Albert; 63.5% reduction for Mkuranga1). Flupyradifurone provided much greater reductions in the number of whitefly nymphs than it did for adults. Averaging the results for 2 MAP to 4 MAP, nymph numbers in the flupyradifurone treatment for Albert were 9.8% of the number in the control, whilst the similar comparison for adults was 38%. Similarly, for Mkuranga1, the values were 11% for nymphs versus 49% for adults.

**Figure 5:**
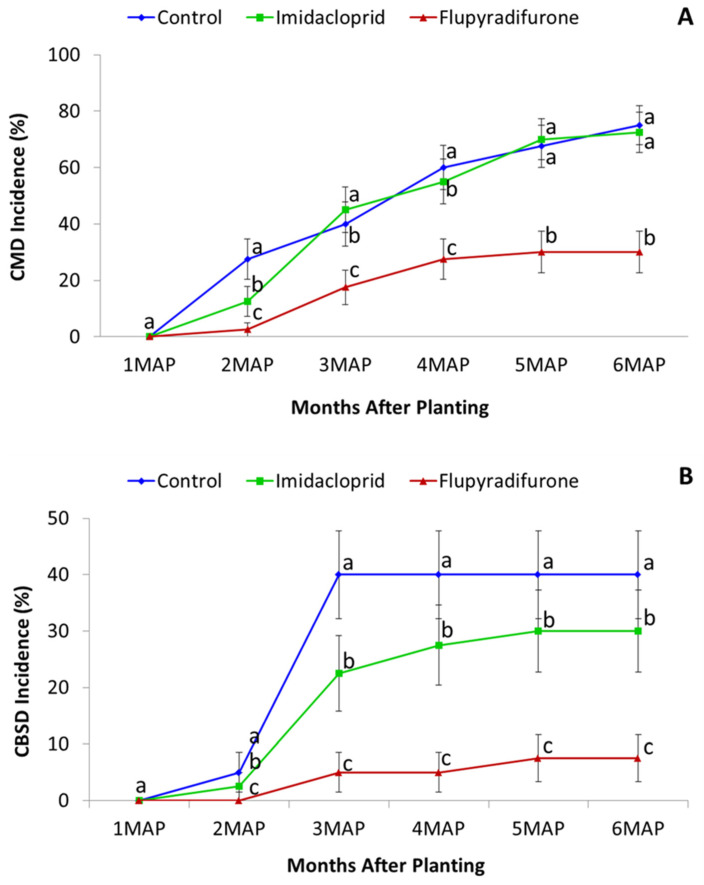
Cassava mosaic disease incidence for cutting dip treatments (flupyradifurone and imidacloprid) of variety Albert (**A**) and cassava brown streak incidence of variety Mkuranga1 (**B**). MAP—Months after planting (Means ± SE). Means with same letter within MAP are not significantly different (*p* < 0.05, Tukey–Kramer).

CMD incidence was significantly lower (*p* < 0.05) in Albert treated with flupyradifurone compared to the control for the six-month duration, except at 1 MAP, by which time no infection had been recorded in any of the plots (Figure 5A). CMD was very low for all treatments of Mkuranga1 throughout the experiment and there were no differences in CMD incidence between treated and control plots (Table 7). CBSD incidence for Albert was significantly lower (*p* < 0.05) with flupyradifurone compared to the control at 2 MAP only, which corresponded to the time where the first infections of CBSD were recorded in the experiment. For CBSD-resistant Mkuranga1, the flupyradifurone treatment had significantly less CBSD infection than the control for the second half of the experiment from 3 MAP to 6 MAP (Table 8; Figure 5B).

#### 3.2.3. Effect of Foliar Insecticide Application on Root Yield

**Figure 6:**
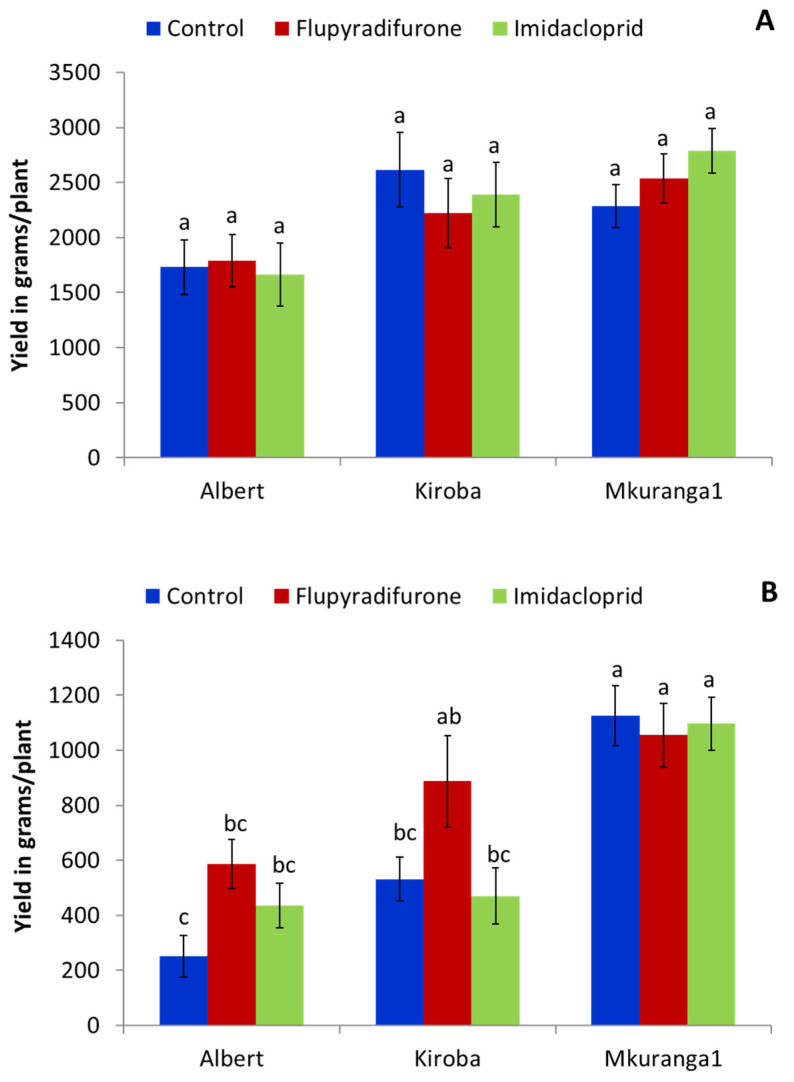
Yield of three cassava varieties (Albert, Kiroba, Mkuranga1) in response to flupyradifurone and imidacloprid insecticides spray treatments for Masika 2017 (**A**) and Vuli 2017 (**B**) (Means ± SE). Means with same letter are not significantly different (*p* < 0.05, Tukey–Kramer test).

There were no significant differences in cassava tuberous root yield between the three insecticide treatments for the Masika season (Figure 6A). The analysis of Masika yield for variety alone revealed significantly lower yield (*p* = 0.003) for Albert compared to Kiroba and Mkuranga1. In the Vuli season, average yields were greater in the flupyradifurone treatment, but differences were not statistically significant (Figure 6B). The analysis of Vuli yield for variety alone revealed a significantly lower yield (*p* < 0.0001) for Albert compared to Kiroba and Mkuranga1.

#### 3.2.4. Effect of Cutting Dip Insecticide Application on Root Yield

**Figure 7:**
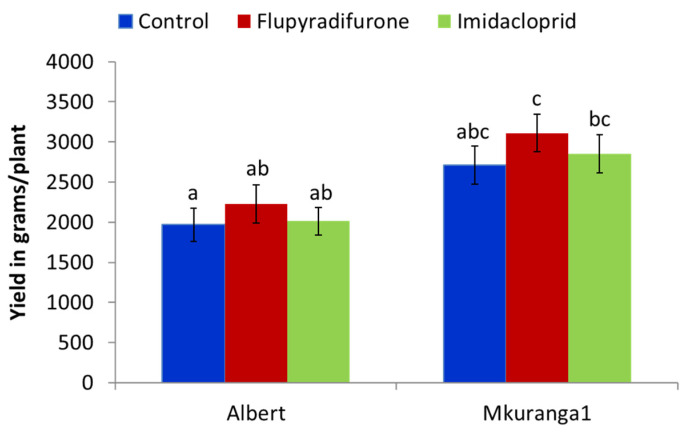
Yield of two cassava varieties (Albert, Mkuranga1) in response to cutting dipping in flupyradifurone and imidacloprid insecticides (Means ± SE). Means with the same letter are not significantly different (*p* < 0.05, Tukey–Kramer test).

Yields for both Albert and Mkuranga1 were 13–14% higher in the flupyradifurone treatment compared to respective controls; however, these differences were not statistically significant (Figure 7).

## 4. Discussion

The whitefly *Bemisia tabaci* is polyphagous pest that causes damage to numerous crops through direct feeding and indirectly through transmission of plant viruses. The major control measure employed against the insect and reduction in virus damage in most crop production systems is the use of chemical pesticides. The occurrence of cryptic species that are known to differ in host plant and chemical response, and rapid insecticide resistance development to existing chemistries necessitates constant evaluation of novel pesticides against this pest [33]. This study evaluated for the first time the efficacy of flupyradifurone (Sivanto SL 200), a novel chemistry pesticide belonging to the chemical class of butenolides, against cassava *Bemisia tabaci* haplogroup SSA-ESA (mitotype SSA1-SG3), which is one of the cryptic species that specializes on cassava [31,32]. We employed a range of lab- and field-based approaches to evaluate the effectiveness of flupyradifurone in controlling cassava whiteflies and made comparisons with a current industry standard for sucking pests—the neonicotinoid imidacloprid.

The leaf disc assay and LC_50_ tests showed that flupyradifurone caused high levels of mortality compared to untreated controls even at the lowest concentration tested, although this activity was not significantly different to that recorded for imidacloprid. Flupyradifurone caused 70% mortality within 24 h under laboratory conditions (leaf dip assays), rising to 96% after 96 h. Comparable results were obtained for imidacloprid (ranging from 63–92%). This mortality range for flupyradifurone is comparable to what has been reported in other studies that used different cryptic species that were in the approximate ranges of 60–95% [25,37]. LC_50_ values recorded in our experiments for flupyradifurone (12.7 g a.i/100 L) and imidacloprid (12.6 g a.i/100 L) are slightly higher than those recorded during tests of other whitefly-control products [38,39], but this is in part a result of the longer durations used between application and mortality recording for these two studies and different whitefly stages considered.

Screenhouse cutting dip experiments demonstrated high levels of whitefly mortality for plants generated from cuttings soaked for 60 min as opposed to 15 and 30 min. An average of 75% fewer whiteflies settled on flupyradifurone-treated plants compared to the control within 1 day of introduction, and no whiteflies settled on subsequent days for up to 5 days. Imidacloprid had 36% fewer whiteflies settling compared to the control within 1 day and still had whiteflies present on treated plants up to day 5. These findings demonstrate that flupyradifurone is systemically active when administered through soaking for a longer duration than imidacloprid. Field studies testing efficacy through spraying demonstrated large contrasts in whitefly abundances between seasons, and consequently different patterns of effectiveness for application of the two insecticides. There were few whiteflies during the long ‘Masika’ rainy season (March planting), and insecticide treatment effects were therefore not pronounced. This was also accompanied by very low incidences of CMD (<12%), which were observed only in the control treatment of the most susceptible variety Kiroba. Similarly, the highest CBSD incidence of 26% was recorded from the control treatment of the CBSD-susceptible variety Albert. Variety Mkuranga1 did not have any symptoms of CMD or CBSD during the Masika season. Because of low whitefly abundances and similarly low incidences of diseases caused by whitefly-transmitted viruses, there were no measurable yield benefits accrued in the plots treated with either of the two insecticides. The absence of either CMD or CBSD in any treatments of variety Mkuranga1 also highlights the importance of virus resistance as a strategy for managing whitefly-borne viruses.

There were greater abundances of whiteflies during the Vuli season. This included significantly more *B. tabaci* adults on Kiroba and Albert in the untreated control than on Mkuranga1 from 2–4 MAP. Similarly, in the cutting dip experiment, abundances of whitefly adults in the control treatment were greater on Albert than those on Mkuranga1 from 1–3 MAP, and there were more nymphs on Albert than on Mkuranga1 at 1 MAP and 6 MAP. These results suggest that Mkuranga1 is less suitable as a host for *B. tabaci* than other cassava varieties, an observation that confirms results obtained from a study in coastal Tanzania that did not include any whitefly-control measures, in which Mkuranga1 had the lowest whitefly adult abundance of the seven varieties evaluated [40]. The performance of Mkuranga1 suggests that resistant or tolerant varieties have an important role to play in management of both vectors and viruses, as noted elsewhere [34,41,42,43,44].

Both insecticidal treatments were effective in reducing whitefly populations in each of the spray and cutting experiments. Differences between whitefly abundance in insecticide-treated and control plots were greatest during the earlier assessments (1 MAP and 2 MAP), which fell shortly after spray treatments. The knockdown assessments at 3 WAP and 6 WAP demonstrated the immediate potency of both imidacloprid and flupyradifurone, as whitefly abundances were reduced to close to zero after 24 h, whilst numbers in the control treatment increased. This result contrasts with the pattern observed in the 3 MAP and 4 MAP datasets in which there were generally no significant differences in abundance between control and insecticide treatments. Whiteflies are known to have a high degree of mobility within cassava fields [45,46]. Therefore, as insecticidal activity declines over time, it would be expected that short-distance whitefly adult movements between plots would lead to gradual reductions in whitefly abundance differences between plots.

The effectiveness of insecticidal treatments in controlling *B. tabaci* whiteflies was reflected in reductions in incidence of whitefly-borne virus disease in both the Vuli spray trial and the cutting dip field experiment. These effects varied greatly, however, depending on the season, variety, and insecticide. Virus incidence reductions caused by insecticidal treatment were most apparent in the Vuli season (for both the spray and cutting dip trials) where whitefly abundances were higher. During Masika, when whitefly populations were low, there was little virus incidence, even in the control treatment. In the cutting dip trial, CBSD disease pressure coupled with high whitefly abundance was such that even insecticide-treated plots had incidences > 90% by 6 MAP. These patterns were also reflected in yield differences. Although there were no statistically significant yield differences between treatments in any of the three field trials, greatest differences in yields (for varieties Albert and Kiroba) occurred in the Vuli spray trial where virus disease incidence differences between treatments were greatest. Contrasts between the high virus disease pressure of the Vuli season and the low pressure in the Masika season were previously described by [40]. This finding was used as the basis for a recommendation to encourage more farmers in coastal Tanzania to plant during the Masika season. The study by [40] also highlighted the important and consistent difference in whitefly abundances between the Vuli (high abundance) and Masika (low abundance) seasons.

Flupyradifurone has been shown to be effective in controlling whitefly-transmitted viruses in other crop systems. For example, Tomato yellow leaf curl virus (TYLCV) infection levels were reduced by up to 85% in tomato [25], Cucurbit yellow stunting disorder virus (CYSDV) transmission and spread were reduced in cantaloupe under greenhouse and field conditions [47], and reductions in Tomato chlorosis virus (ToCV) transmission in flupyradifurone-treated tomatoes were attributed to reduced phloem activity of the virus [48].

The superior performance of flupyradifurone in controlling cassava whiteflies when compared to imidacloprid, particularly in the cutting dip experiments, suggest that it has better systemic activity. Although no known studies are reported on the efficacy of flupyradifurone when used to dip planting material for propagation, a study with the neonicotinoids thiamethoxam and acetamiprid showed that this method is effective at controlling *B. tabaci* whiteflies on poinsettia [49]. Whitefly abundances were reduced by 62–88%, although there was no significant difference between foliar spraying and cutting immersion application methods [49]. A study evaluating efficacy of various insecticides including flupyradifurone using foliar application and root drenching also showed no significant differences in whitefly mortality between the methods [37]. A striking feature of our evaluation of flupyradifurone for cassava, however, is that a cutting single dip application prior to planting delivered sustained reductions in the abundance of whitefly adults and nymphs up to six months after planting, which in turn resulted in a 60% reduction in CMD incidence for variety Albert and an 81% reduction in CBSD incidence for Mkuranga1. In view of these promising station-based experimental results, there is clear value in testing this whitefly-control approach under on-farm conditions. To obtain the most realistic results, this would need to be conducted by comparing farms fully treated with flupyradifurone with untreated farms. The cutting dipping application of flupyradifurone, if effective under field conditions, could then be widely promoted to reduce cost and to avoid the deleterious effects that direct spraying has on non-target organisms. Although many smallholder cassava farmers in sub-Saharan Africa affected by whitefly-transmitted viruses primarily produce for subsistence purposes, there is an increasing number of commercially oriented producers. Applying effective whitefly control measures such as the systemic application of flupyradifurone or similarly effective insecticides may represent a worthwhile investment as part of a broader IPM strategy also including the purchase of certified planting material of disease tolerant varieties. Although the Masika season has low whitefly numbers, timing of planting may contribute less to control of cassava *B. tabaci*, as the majority of farmers plant the crop during the Vuli season. This is attributed to cropping systems where the Masika season is dedicated primarily to cereal crops such as maize.

## 5. Conclusions

This study presents the first results evaluating the efficacy of the novel butenolide insecticide flupyradifurone (Sivanto SL 200) against cassava-colonizing *Bemisia tabaci* SSA-ESA (SSA-SG3) through a standard spray method and a novel cutting absorption approach. Flupyradifurone caused significant whitefly mortality under laboratory, screenhouse and field conditions in comparison to the standard insecticide imidacloprid and control. Cutting dip treatment with flupyradifurone provided sustained reductions in the abundance of both nymph and adult whitefly stages for up to six months. Reductions in whitefly abundance achieved through cutting dipping resulted in lower incidences of CMD for the CMD-susceptible Kiroba variety and reduced levels of CBSD infection for variety Mkuranga1 under high disease pressure conditions. Differences demonstrated in the relative susceptibility of varieties tested to whiteflies and the viruses that they transmit confirmed that deploying sources of resistance to both whiteflies and whitefly-transmitted viruses should continue to be key tactics for the management of these virus-vector complexes. Furthermore, the findings from this study demonstrate that cutting dips using flupyradifurone could be incorporated into an IPM package for reducing whitefly populations and virus incidence in cassava. This could be particularly important for the increasing number of commercial cassava planting material producers whose businesses depend on maintaining CBSD and CMD levels below prescribed levels for certification.

## Figures and Tables

**Table 1:** Effects of cutting dips of varying length and with two insecticides or water in causing mortality in adult *B. tabaci* whiteflies placed on newly sprouted plants derived from those cuttings. Results presented are mean whitefly abundances per plant ± SE ^Z^.

Dipping Duration	Treatment	Number of Whiteflies				
		Day 1	Day 2	Day 3	Day 4	Day 5
15 min	Control	29.8 ± 1.3 c	28.9 ± 2.4 d	27.8 ± 2.5 d	26.7 ± 2.6 d	25.8 ± 2.4 c
	Flupyradifurone	26.9 ± 1.4 c	18.8 ± 1.8 c	10.8 ± 1.5 c	1.7 ± 0.6 b	0.3 ± 0.2 a
	Imidacloprid	27.8 ± 3.0 c	25.6 ± 3.3 cd	20.0 ± 2.0 c	9.7 ± 2.2 c	3.5 ± 1.2 b
30 min	Control	30.0 ± 3.6 c	30.0 ± 3.0 d	29.8 ± 3.0 d	29.8 ± 2.6 d	28.6 ± 2.5 c
	Flupyradifurone	30.0 ± 0.8 c	22.3 ± 3.1 c	5.9 ± 2.6 b	0.0 ± 0.0 a	0.0 ± 0.0 a
	Imidacloprid	30.0 ± 1.9 c	24.1 ± 2.2 cd	14.5 ± 2.8 c	1.8 ± 1.7 b	0.0 ± 0.0 a
60 min	Control	29.3 ± 1.2 c	29.2 ± 0.7 d	28.4 ± 1.1 d	27.6 ± 1.3 d	25.8 ± 3.0 c
	Flupyradifurone	7.3 ± 2.8 a	0.0 ± 0.0 a	0.0 ± 0.0 a	0.0 ± 0.0 a	0.0 ± 0.0 a
	Imidacloprid	18.7 ± 2.4 b	11.2 ± 1.4 b	9.2 ± 1.7 b	2.9 ± 1.0 b	1.3 ± 0.6 a
	*p* value	<0.0001	<0.0001	<0.0001	<0.0001	0.0466

^Z^ Means with the same letter within a column are not significantly different (*p* < 0.05, Tukey–Kramer test).

**Table 2:** Whitefly abundance for Vuli 2017 in the foliar application trial (Means ± SE) ^Z^.

Variety	Treatment	Number of Adult Whiteflies					
		1 MAP	2 MAP	3 MAP	4 MAP	5 MAP	6 MAP
Albert	Control	1.6 ± 0.4 ed	10.3 ± 1.7 e	29.0 ± 3.5 d	14.9 ± 1.9 g	0.0 ± 0.0	0.0 ± 0.0
	Flupyradifurone	0.5 ± 0.2 a	0.1 ± 0.1 ab	30.8 ± 2.7 d	9.6 ± 1.1 f	0.0 ± 0.0	0.0 ± 0.0
	Imidacloprid	1.2 ± 0.3 d cb	0.5 ± 0.1 b	31.8 ± 3.4 d	6.3 ± 0.8 d	0.0 ± 0.0	0.0 ± 0.0
Kiroba	Control	2.1 ± 0.4 e	4.4 ± 0.8 d	23.8 ± 2.4 c	7.8 ± 1.2 def	0.0 ± 0.0	0.0 ± 0.0
	Flupyradifurone	0.6 ± 0.2 ab	0.1 ± 0.0 ab	22.0 ± 2.6 c	6.7 ± 1.2 de	0.0 ± 0.0	0.0 ± 0.0
	Imidacloprid	1.5 ± 0.4 edc	0.5 ± 0.2 b	28.7 ± 2.8 d	8.2 ± 1.2 ef	0.0 ± 0.0	0.0 ± 0.0
Mkuranga1	Control	1.9 ± 0.3 ed	1.5 ± 0.3 c	6.6 ± 0.9 b	3.8 ± 0.8 c	0.0 ± 0.0	0.0 ± 0.0
	Flupyradifurone	0.9 ± 0.2 cba	0.0 ± 0.0 a	5.9 ± 0.9 ab	1.1 ± 0.4 a	0.0 ± 0.0	0.0 ± 0.0
	Imidacloprid	0.7 ± 0.1 ab	0.1 ± 0.1 ab	4.6 ± 0.9 a	2.2 ± 0.6 b	0.0 ± 0.0	0.0 ± 0.0
		*p* = 0.0008	*p* = 0.0287	*p* < 0.0001	*p* < 0.0001	-	-

^Z^ Means with same letter within column are not significantly different (*p* < 0.05, Tukey–Kramer test).

**Table 3:** Cassava mosaic disease (CMD) percentage incidence for Vuli 2017 (Means ± SE) ^Z^.

Variety	Treatment	CMD Incidence					
		1 MAP	2 MAP	3 MAP	4 MAP	5 MAP	6 MAP
Albert	Control	0.0 ± 0.0 a	8.3 ± 4.0 a	8.3 ± 4.0 ab	8.3 ± 4.0 ab	8.3 ± 4.0 ab	8.3 ± 4.0 ab
	Flupyradifurone	3.8 ± 2.7 a	3.9 ± 2.7 a	3.9 ± 2.7 a	12.0 ± 4.6 ab	12.0 ± 4.6 ab	12.0 ± 4.6 ab
	Imidacloprid	1.8 ± 1.8 a	3.8 ± 2.7 a	3.8 ± 2.7 a	3.9 ± 2.7 ab	3.9 ± 2.7 ab	10.6 ± 4.5 ab
Kiroba	Control	0.0 ± 0.0 a	6.3 ± 3.5 a	13.0 ± 5.0 ab	21.7 ± 6.1 b	25.6 ± 6.7 b	25.6 ± 6.7 bc
	Flupyradifurone	2.2 ± 2.2 a	2.7 ± 2.7 a	11.4 ± 5.5 ab	11.8 ± 5.6 ab	15.2 ± 6.3 ab	15.2 ± 6.3 abc
	Imidacloprid	2.0 ± 2.0 a	4.4 ± 3.1 a	22.7 ± 6.4 b	25.0 ± 6.6 b	26.2 ± 6.9 b	29.3 ± 7.2 c
Mkuranga1	Control	0.0 ± 0.0 a	1.8 ± 1.8 a	1.8 ± 1.8 a	1.8 ± 1.8 a	1.8 ± 1.8 a	1.8 ± 1.8 a
	Flupyradifurone	0.0 ± 0.0 a	0.0 ± 0.0 a	0.0 ± 0.0 a	0.0 ± 0.0 a	0.0 ± 0.0 a	0.0 ± 0.0 a
	Imidacloprid	0.0 ± 0.0 a	0.0 ± 0.0 a	0.0 ± 0.0 a	0.0 ± 0.0 a	0.0 ± 0.0 a	0.0 ± 0.0 a
	*p* value	0.7240	0.9680	<0.0001	< 0.0001	<0.0001	<0.0001

^Z^ Means with same letter within column are not significantly different (*p* = 0.05, Tukey–Kramer).

**Table 4:** Cassava brown streak disease (CBSD) percentage incidence for Vuli 2017 (Means ± SE) ^Z^.

Variety	Treatment	CBSD Incidence					
		1 MAP	2 MAP	3 MAP	4 MAP	5 MAP	6 MAP
Albert	Control	0.0 ± 0.0	0.0 ± 0.0 a	14.6 ± 5.1 ab	60.4 ± 7.1 d	83.3 ± 5.4 d	85.4 ± 5.1 d
	Flupyradifurone	0.0 ± 0.0	0.0 ± 0.0 a	13.7 ± 4.9 ab	22.0 ± 5.9 bc	28.0 ± 6.4 b	38.0 ± 6.9 bc
	Imidacloprid	0.0 ± 0.0	0.0 ± 0.0 a	19.2 ± 5.5 b	39.2 ± 6.9 cd	54.9 ± 7.0 c	59.6 ± 7.2 c
Kiroba	Control	0.0 ± 0.0	0.0 ± 0.0 a	15.2 ± 5.4 ab	23.9 ± 6.4 bc	37.2 ± 7.5 bc	41.9 ± 7.6 bc
	Flupyradifurone	0.0 ± 0.0	2.7 ± 2.7 a	8.6 ± 4.8 ab	11.8 ± 5.6 ab	12.1 ± 5.8 ab	18.2 ± 6.8 ab
	Imidacloprid	0.0 ± 0.0	2.2 ± 2.2 a	13.6 ± 5.2 ab	18.2 ± 5.9 abc	28.6 ± 7.1 b	29.3 ± 7.2 b
Mkuranga1	Control	0.0 ± 0.0	0.0 ± 0.0 a	0.0 ± 0.0 a	0.0 ± 0.0 d	0.0 ± 0.0 a	0.0 ± 0.0 a
	Flupyradifurone	0.0 ± 0.0	0.0 ± 0.0 a	0.0 ± 0.0 a	0.0 ± 0.0 d	0.0 ± 0.0 a	0.0 ± 0.0 a
	Imidacloprid	0.0 ± 0.0	0.0 ± 0.0 a	0.0 ± 0.0 a	0.0 ± 0.0 d	0.0 ± 0.0 a	0.0 ± 0.0 a
	*p* value	-	0.5664	<0.0001	0.0022	<0.0001	0.0002

^Z^ Means with same letter within column are not significantly different (*p* < 0.05, Tukey–Kramer).

**Table 5:** Abundance of adult whitefly in the cutting dip experiment, 2020 (Means ± SE) ^Z^.

Variety	Treatment	Number of Adult Whiteflies					
		1 MAP	2 MAP	3 MAP	4 MAP	5 MAP	6 MAP
Albert	Control	11.8 ± 1.5 d	27.5 ± 5.4 e	12.2 ± 2.7 c	3.8 ± 0.6 b	13.2 ± 1.8 c	15.9 ± 2.6 d
	Flupyradifurone	2.4 ± 0.5 ab	4.9 ± 0.9 a	6.6 ± 1.7 a	1.6 ± 0.5 a	3.3 ± 0.7 a	6.7 ± 1.1 c
	Imidacloprid	3.4 ± 0.7 b	17.8 ± 2.8 d	12.8 ± 2.3 c	2.7 ± 0.6 b	6.8 ± 1.1 b	13.5 ± 2.2 d
Mkuranga1	Control	4.7 ± 0.8 c	14.2 ± 2.7 c	8.6 ± 2.0 b	5.5 ± 1.0 c	7.0 ± 1.2 b	5.8 ± 1.2 b
	Flupyradifurone	1.9 ± 0.4 a	6.8 ± 1.1 b	6.1 ± 1.1 a	1.5 ± 0.4 a	2.8 ± 0.7 a	4.1 ± 0.7 a
	Imidacloprid	1.8 ± 0.5 a	15.9 ± 2.9 cd	6.0 ± 1.3 a	2.8 ± 0.6 b	5.7 ± 1.0 b	8.2 ± 1.3 c
	*p* value	0.0008	<0.0001	<0.0001	0.0300	<0.0001	<0.0001

^Z^ Means with same letter within column are not significantly different (*p* < 0.05, Tukey–Kramer test).

**Table 6:** Whitefly nymph abundance for cutting dipping method, 2020 (Means ± SE) ^Z^.

Variety	Treatment	Number of Nymphs					
		1 MAP	2 MAP	3 MAP	4 MAP	5 MAP	6 MAP
Albert	Control	3.5 ± 0.8 c	43.4 ± 6.3 d	28.7 ± 7.3 d	22.6 ± 3.9 d	7.3 ± 2.0 d	12.8 ± 2.8 d
	Flupyradifurone	1.2 ± 0.3 a	4.0 ± 0.9 a	3.0 ± 1.4 a	2.5 ± 1.0 a	2.0 ± 1.0 a	5.3 ± 1.1 b
	Imidacloprid	2.7 ± 0.7 bc	17.2 ± 2.8 b	13.2 ± 2.9 b	6.4 ± 1.6 b	2.9 ± 1.0 c	5.0 ± 0.9 b
Mkuranga1	Control	2.2 ± 0.8 b	42.3 ± 6.1 d	30.1 ± 5.3 d	24.1 ± 3.8 d	6.9 ± 2.3 d	7.8 ± 1.6 c
	Flupyradifurone	1.1 ± 0.3 a	4.5 ± 1.3 a	4.2 ± 1.1 a	2.0 ± 0.9 a	0.5 ± 0.2 a	2.0 ± 0.7 a
	Imidacloprid	1.2 ± 0.4 a	20.7 ± 4.3 c	16.4 ± 3.6 c	8.8 ± 1.6 c	4.0 ± 1.5 c	4.4 ± 1.0 b
	*p* value	0.0176	0.0025	0.00156	0.0025	<0.0001	<0.0001

^Z^ Means with same letter within column are not significantly different (*p* < 0.05, Tukey–Kramer test).

**Table 7:** Cassava mosaic disease (CMD) percentage incidence for cutting dipping method, 2020 (Means ± SE) ^Z^.

Variety	Treatment	CMD Incidence					
		1 MAP	2 MAP	3 MAP	4 MAP	5 MAP	6 MAP
Albert	Control	0.0 ± 0.0	27.5 ± 7.1 b	40.0 ± 7.8 b	60.0 ± 7.8 c	67.5 ± 07.5 c	75.0 ± 6.9 c
	Flupyradifurone	0.0 ± 0.0	2.5 ± 2.5 a	17.5 ± 6.1 a	27.5 ± 7.1 b	30.0 ± 7.3 b	30.0 ± 7.3 b
	Imidacloprid	0.0 ± 0.0	12.5 ± 0.1 ab	45.0 ± 8.0 b	55.0 ± 8.0 c	70.0 ± 7.3 c	72.5 ± 7.1 c
Mkuranga1	Control	0.0 ± 0.0	0.0 ± 0.0 a	0.0 ± 0.0 a	0.0 ± 0.0 a	0.0 ± 0.0 a	0.0 ± 0.0 a
	Flupyradifurone	0.0 ± 0.0	0.0 ± 0.0 a	5.0 ± 3.5 a	5.0 ± 3.5 bc	5.0 ± 3.5 a	5.0 ± 0.0 a
	Imidacloprid	0.0 ± 0.0	0.0 ± 0.0 a	0.0 ± 0.0 a	0.0 ± 0.0 a	0.0 ± 0.0 a	0.0 ± 0.0 a
	*p* value	-	0.0044	0.0057	0.0016	<0.0001	<0.0001

^Z^ Means with same letter within column are not significantly different (*p* < 0.05, Tukey–Kramer test).

**Table 8:** Cassava brown streak disease (CBSD) percentage incidence for cutting dipping method, 2020 (Means ± SE) ^Z^.

Variety	Treatment	CBSD Incidence					
		1 MAP	2 MAP	3 MAP	4 MAP	5 MAP	6 MAP
Albert	Control	0.0 ± 0.0	52.5 ± 8.0 b	92.5 ± 4.2 c	95.0 ± 3.5 c	97.5 ± 2.5 c	97.5 ± 2.5 c
	Flupyradifurone	0.0 ± 0.0	15.0 ± 5.7 a	87.5 ± 5.3 c	90.0 ± 4.8 c	100 ± 0.0 c	100 ± 0.0 c
	Imidacloprid	0.0 ± 0.0	42.5 ± 7.9 b	82.5 ± 6.1 c	90.0 ± 4.8 c	97.5 ± 2.5 c	100 ± 0.0 c
Mkuranga1	Control	0.0 ± 0.0	5.0 ± 3.5 a	40.0 ± 7.8 b	40.0 ± 7.8 b	40.0 ± 7.8 b	40.0 ± 7.8 b
	Flupyradifurone	0.0 ± 0.0	0.0 ± 0.0 a	5.0 ± 3.5 a	5.0 ± 3.5 a	7.5 ± 4.2 a	7.5 ± 4.2 a
	Imidacloprid	0.0 ± 0.0	2.5 ± 2.5 a	22.5 ± 6.7 ab	27.5 ± 7.1 b	30.0 ± 7.3 b	30.0 ± 7.3 b
	*p* value	-	0.0083	0.0279	0.0197	0.0015	0.0014

^Z^ Means with same letter within column are not significantly different (*p* < 0.05, Tukey–Kramer test).

## Data Availability

All data is provided in the manuscript.
